# Cholesterol in the Pathogenesis of Alzheimer’s, Parkinson’s Diseases and Autism: Link to Synaptic Dysfunction

**Published:** 2017

**Authors:** A. M. Petrov, M. R. Kasimov, A. L. Zefirov

**Affiliations:** Kazan State Medical University, Normal Physiology department, Butlerova str. 49, Kazan, 420012, Russia

**Keywords:** cholesterol, oxysterols, lipid rafts, neurodegenerative disease, synaptic transmission

## Abstract

In our previous review, we described brain cholesterol metabolism in control conditions and in the case of some rare neurological pathologies linked to defects in the genes which are directly involved in the synthesis and/or traffic of cholesterol. Here, we have analyzed disruptions in cholesterol homeostasis in widespread neurodegenerative diseases (Alzheimer’s and Parkinson’s diseases) and autism spectrum disorders. We particularly focused on the synaptic dysfunctions that could arise from changes in both membrane cholesterol availability and oxysterol production. Notably, alterations in the brain cholesterol metabolism and neurotransmission occur in the early stages of these pathologies and the polymorphism of the genes associated with cholesterol homeostasis and synaptic communication affects the risk of onset and severity of these diseases. In addition, pharmacological and genetic manipulations of brain cholesterol homeostasis in animal models frequently have marked effects on the progression of neurodegenerative diseases. Thus, the development of Alzheimer’s, Parkinson’s and autism spectrum disorders may be partially associated with an imbalance of cholesterol homeostasis that leads to changes in the membrane cholesterol and oxysterol levels that, in turn, modulates key steps in the synaptic transmission.

## INTRODUCTION

Previously, we described changes in cholesterol metabolism in rare hereditary pathologies of the central nervous system caused by mutations in the genes which are directly involved in the biosynthesis of cholesterol (Smith-Lemli-Opitz syndrome) or its intracellular traffic (Niemann-Pick type C disease) and synthesis regulation (Huntington disease) [1]. In this review, we analyze the relationships between such common neurodegenerative diseases as Alzheimer’s and Parkinson’s and autism spectrum disorders and cholesterol homeostasis and synaptic dysfunction.

## ALZHEIMER’S DISEASE (AD)

**Fig. 1:**
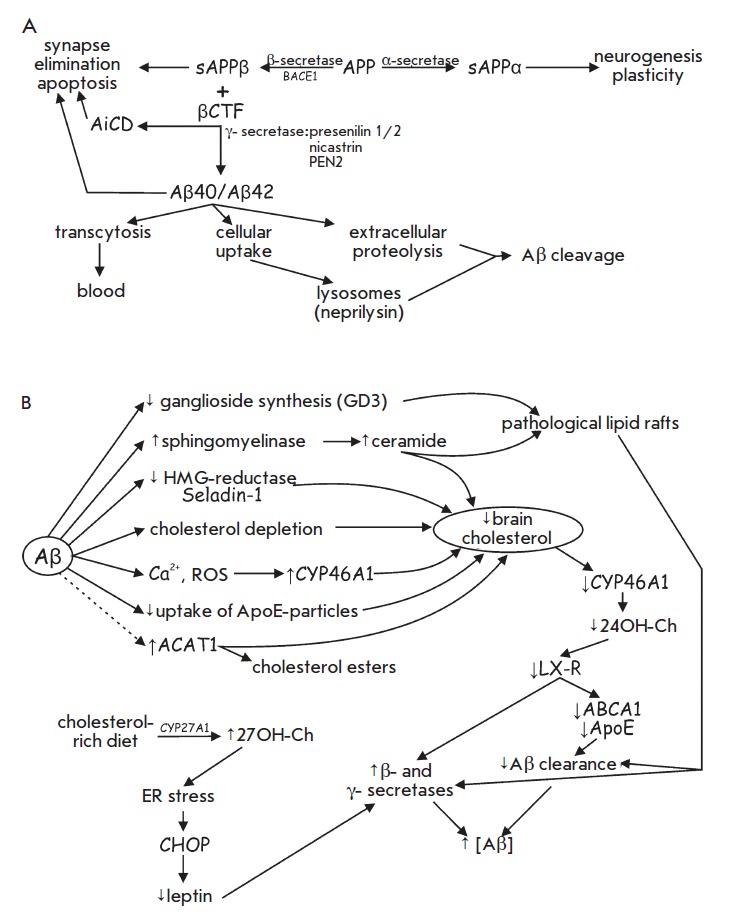
Synthesis of amyloid peptide and brain level of cholesterol. A – Amyloid precursor protein (APP) processing: involvement of α-, β- and γ-secretases. B – Links between amyloid peptide β (Aβ) production and brain cholesterol metabolism. Impact of cholesterol-rich diet. See text for details.

Alzheimer’s disease is the most widespread neurodegenerative disease. It usually affects older people and manifests itself as a deterioration of memory and cognitive abilities. It involves the deposition of amyloid peptide β (Aβ) into amyloid plaques in the extracellular space of the brain and neurofilament bundles of the hyperphosphorylated tau protein inside cells. An accumulation of Aβ and death of neurons, especially in the hippocampus, are considered the main manifestations of AD. The accumulation of Aβ reflects the imbalance between its production and its elimination from the brain. Aβ is formed in a two-step cleavage of the transmembrane protein APP (amyloid precursor protein) by proteases called secretases. The APP is first cleaved by either secretase α or β, and then by γ. The cleavage of APP by α-secretase results in a nonamyloidogenic product, sAPPα, which does not cause the disease. Moreover, sAPPα has neuroprotective effects and enhances long-term potentiation and learning ability. APP is cleaved by β-secretase (BACE1) to release the soluble fragment sAPPβ (involved in the elimination of synapses and apoptosis) and a C-terminal fragment (βCTF) which is subsequently cut by γ-secretase (a complex of several proteins comprising presenilin 1 or 2, APH, nicastrin and PEN2) with the formation of toxic Aβ containing 40 or 42 (more toxic) amino acid residues. In addition, βCTF proteolysis by γ-secretase results in the release of the intracellular APP domain (AICD), which, in cooperation with Fe65 and TIP60, can trigger the transcription of genes which accelerate cell death and disrupt neurogenesis [2]. Aβ in the brain interstitial fluid can be removed via several mechanisms: transfer through the BBB, uptake by cells for degradation in the lysosomes, and cleavage by specific proteases (*Fig. 1A*). Lysosomes contain a specific Aβ-degrading protease, neprilysin, whereas outside the cell Aβ clearance is performed by an insulin-degrading enzyme (IDE) which is secreted by astrocytes and microglia [3]. There are two forms of AD: with early (5–10%) and late (90–95%) onset in which the disease symptoms appear before and after 65 years of age, respectively. The early form is strictly hereditary and is associated with excessive production of Aβ. Patients with a late onset type of AD usually have inefficient Aβ clearance rather than an enhanced production [4, 5].

**The levels of cholesterol in the brain and blood plasma: correlations with AD, role of oxysterols**

Experimental data support the significant contribution of changes in cholesterol metabolism to the pathogenesis of AD (*Fig. 1B*). However, it is unclear whether the disruption in cholesterol homeostasis is a cause or a consequence of the disease. An earlier work revealed Aβ accumulation in the brain of rabbits receiving a cholesterol-rich diet [6]. Later research showed that cholesterol intake increases tau phosphorylation, causes oxidative stress and cognitive defects, but does not change the level of cholesterol in a rabbit’s brain [7]. Several epidemiological studies (but not all of them) found an increased risk of AD in individuals with elevated plasma cholesterol, especially in mid-life [2, 8]. There is a confirmed correlation between high (or low) cholesterol content in low (high) density lipoproteins and Aβ levels in the brain of patients in early stages of AD [9]. However, the relationship between cholesterol levels in plasma and AD may be mediated by changes in the vascular tone and inflammatory responses rather than by direct influence on the brain cholesterol. Chronic cerebral hypoperfusion in rats and mice with AD increases BACE1 expression, Aβ concentration, and cognitive defects [10]. Hypoperfusion and inflammation, high levels of cholesterol in the plasma can cause AD-promoting vascular dysfunction and changes in oxysterol production.

In AD, the concentration of 24S-hydroxycholesterol (24OH-Ch) decreases in the brain. However, in patients at the initial stages of AD the level of 24OH-Ch transiently increases in the plasma and cerebrospinal fluid [11]. Individuals with elevated plasma 24OH-Ch have a higher probability of developing cognitive impairment over the next 8 years [12]. The excessive production of 24OH-Ch may be an indication of an attempt to compensate for the nascent dysfunction [1]. Elevated expression of the 24OH-Ch-synthesizing enzyme CYP46A1 (using adenoviral therapy) in the brain of APP23-mice significantly reduces Aβ accumulation, gliosis, and cognitive defects [13]. The effect of CYP46A1 activation may be mediated by 24OH-Ch that stimulates LXα and β-receptors, which, in turn, increases the expression of the genes involved in cholesterol synthesis and traffic [1]. Deletion of LXα or β-receptor genes causes age-related neurodegenerative disorders [14]. Conversely, activation of LX-receptors increases Aβ clearance and reverses the memory deficit in transgenic APP/PS1 and APP23 mice, probably by upregulating the levels of ApoE and ABCA1 in the brain [15]. ABCA1 can quickly remove the excess Aβ from the membrane into the extracellular space that protects neurons from the toxic effect of Aβ accumulation [16]. In the endothelial cells of brain capillaries, 24OH-Ch increases Aβ clearance, enhancing the expression of ABCA1, and reduces Aβ production, altering secretase expression [17].

The level of 27OH-Ch in the brain is significantly higher in AD [18]. In rabbits receiving a diet rich in cholesterol, the levels of 24OH-Ch and 27OH-Ch in the plasma increase, whereas the ratio of 24OH-Ch to 27OH-Ch in the brain is lower, which may exacerbate the risk of neurodegeneration. It is assumed that an increased influx of 27OH-Ch into the brain and/or increased extrusion of 24OH-Ch from the brain may underlie the association between a high level of cholesterol in the plasma and AD [11, 19]. Studies of organo-typical brain slices from adult animals have shown that 27OH-Ch increases the levels of Aβ and phosphorylated tau, whereas 24OH-Ch promotes nonamyloidogenic APP processing. Moreover, 24OH-Ch inhibits the toxic effects of 27OH-Ch when these oxysterols are co-applied. 27OH-Ch can cause ER stress, resulting in the activation of the transcription factor CHOP (C/EBPα homologous protein), which suppresses the leptin synthesis that normally reduces BACE1 expression, Aβ production, and tau phosphorylation [19].

Although early studies indicated that the level of brain cholesterol is elevated in patients with AD, other researchers have found that brain cholesterol synthesis and the total pool of cholesterol in the brain decrease [8] (*Fig. 1B*). In AD, the level of cholesterol is reduced in the temporal gyrus, hippocampus, lipid raft fraction in the whole brain, and white matter [20-22]. However, the cholesterol content is increased in the cores of mature amyloid plaques and the nerve terminals enriched with amyloid aggregates [23]. These observations suggest the existence of an Aβ-dependent mechanism of cholesterol removal from the nerve terminal membrane. Other ways of lowering brain cholesterol availability in patients with AD can be associated with: APP/Aβ-dependent supression of cholesterol synthesis by inhibition of 3-hydroxy-3-methylglutaryl-CoA reductase (HMG-reductase) [24]; decrease in the uptake of cholesterol-loaded ApoE-particles under the influence of Aβ [15]; increase in cholesterol oxidation due to enhanced CYP46A1 activity [11]; Aβ-induced modification of lipid rafts [1, 25]. The increase in CYP46A1 activity may be caused by Aβ-induced alterations in Ca^2+^ homeostasis and oxidative stress [11, 26]. The aging process is accompanied by a reduction in the cholesterol content, especially noticeable in regions susceptible to AD, which may be related to an increased CYP46A1 expression/activity and decreased cholesterol synthesis/traffic [26, 27].

The brain biopsies of AD patients revealed an accumulation of cholesterol ester-rich lipid drops in Aβ- positive neurons, and the higher the number of such drops, the greater the concentration of Aβ [28]. Inhibition of the synthesis of cholesterol esters (acetyl-CoA-cholesterol acyltransferase/ACAT1) was accompanied by an increase in 24-OH Ch concentration and a markedly reduced generation of Aβ, plaque formation, and cognitive defects in an animal model of AD [29]. It is possible that the early stages of AD involve an increase in the synthesis of the enzymes responsible for the formation of cholesterol esters [30]. One way to stimulate cholesterol esterification may be by increased production of 25-OH Ch, which is caused by inflammation. In addition, the expression of the CYP7B1 enzyme that metabolizes 25- and 27-OH Ch is downregulated in AD [31].

The brain level of sphingomyelin is reduced in AD patients, and the concentration of ceramides, products of sphingomyelin hydrolysis by sphingomyelinases, is increased. Consequently, normal lipid rafts “dissolve,” cholesterol is released from the membranes, and ceramides aggregate to form large ceramide-enriched lipid platforms, which are involved in the initiation of cell death. Sphingomyelinase activation may occur in the early stages of AD in response to Aβ [32, 33]. The brain concentration of gangliosides (lipid raft components) decreases with aging, but patients with AD show a steeper reduction. Aβ and AICD can inhibit and reduce the expression of the enzymes required for the synthesis of several gangliosides. However, the content of the gangliosides GM1 and GM2 involved in Aβ aggregation increases in the AD brain [21]. The imbalance in ganglioside composition can contribute to the conversion of Aβ into a highly toxic oligomeric form [1, 33].

**Aβ synthesis and cholesterol**

**Fig. 2:**
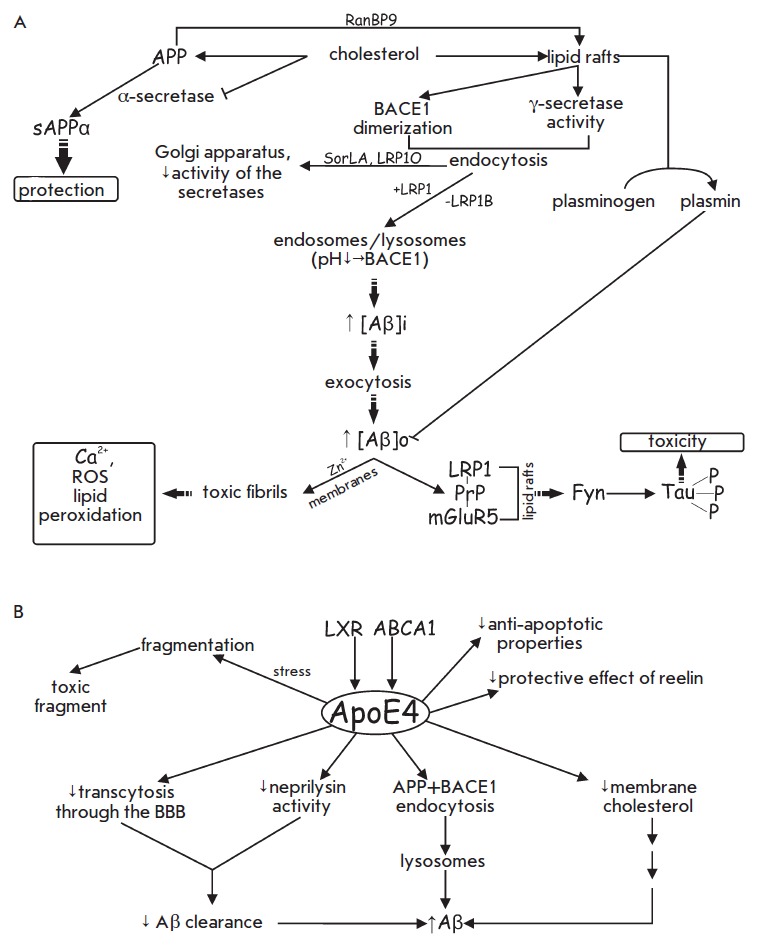
Cholesterol, lipid rafts and ApoE4 in the amyloid peptide β (Aβ) turnover. A – Role of cholesterol and lipid rafts in the production and toxicity of Aβ. B – Influence of ApoE4 on the Aβ synthesis/clearance balance and neuron survival. See text for details.

The extracellular N-terminal fragment of APP contains a cholesterol-binding site [34], but most APP molecules are located outside lipid rafts. Secretases β and γ, which are involved in Aβ synthesis, are both residents of lipid rafts. Expression of the scaffold protein RanBP9 (increased in APP-mice) promotes targeting of the APP to lipid rafts, which contain BACE1 [35]. The activation of β-secretase requires lipid rafts-dependent dimerization and stabilization steps. In the presence of elevated levels of cholesterol and sphingolipis, raft-associated γ-secretase produces a more toxic form, Aβ42. However, the synthesis of Aβ (~ 70%) mainly occurs within a cell [28]. Therefore, it first requires the stage of raft-dependent endocytosis, in which APP, β-, and γ-secretases uptake into a vesicle and are then delivered to endosomes/lysosomes. Aβ is generated in the endolysosomes under acidic conditions that favor BACE1 activity. Subsequently, a portion of Aβ is released into the extracellular space by exocytosis (e.g., as a content of synaptic vesicles) [3] (*Fig. 2A*). Aβ aggregation into toxic oligomers is enhanced by zinc ions from synaptic vesicles [36]. It should be noted that nonamyloidogenic cleavage of APP by α-secretase occurs on the cell surface [3].

The toxic effects of Aβ may depend on its binding to membrane components. In the fibroblasts of AD patients, Aβ interacts with the plasma membrane more actively when the cholesterol level is low, while high levels of cholesterol prevent Aβ-induced generation of reactive oxygen species and lipid oxidation [37]. On the other hand, Aβ-mediated disruption of long-term synaptic potentiation and increased synaptic depression may be caused by its binding to PrP (in this case, metabotropic glutamate receptor 5 and LRP1 act as co-receptors) and the subsequent activation of tyrosine kinase Fyn, which phosphorylates the tau protein. A reduced integrity of rafts due to cholesterol removal disrupts the complex of PrP-metabotropic glutamate receptor 5-LRP1, attenuating the interaction of Aβ with the postsynaptic membranes [8, 38] (*Fig. 2A*).

From one point of view, the increase in membrane cholesterol promotes the association of APP, β-, and γ-secretases within the lipid rafts and increases Aβ production, while other authors suggest that APP and the secretases are distributed into different rafts [3, 34, 39]. A decreased membrane cholesterol content increases APP cleavage by α-secretase, reducing the formation of toxic Aβ [3]. However, activation of the plasminogen into the plasmin, which cuts Aβ, occurs on the surface of the lipid rafts and, therefore, raft disruption can reduce the rate of Aβ degradation [39]. Upon loss of raft integrity, their components, in particular β- and γ-secretases, can diffuse into the liquid-disordered membrane phase, where APP is mainly distributed, and, therefore, Aβ production may increase [32]. AD is associated with a reduced expression of seladin-1 (selective Alzheimer disease indicator 1), a gene that encodes an enzyme converting desmosterol to cholesterol. Deletion of seladin-1 leads to a lower cholesterol level, disorganization of lipid rafts, and Aβ accumulation. Conversely, overexpression of seladin-1 (for example, in response to estrogen administration) accelerates cholesterol metabolism in the brain and increases neurons’ resistance to Aβ [40]. Interestingly, caveolin 1 gene knockout (caveolin 1 stabilizes the membrane rafts) leads to a pathology, similar to that observed in AD, which is accompanied by Aβ accumulation and neurodegeneration. The level of caveolin 1 in neurons reduces with aging, whereas the fluidity of the synaptosomal membranes increases [41]. A low expression of caveolin 1 reduces membrane cholesterol availability, since caveolin 1 is involved in the delivery of newly synthesized cholesterol to the plasma membrane [42].

Some studies indicate that pharmacological strategies for lowering the level of cholesterol in cells, having its initial normal baseline level, inhibit Aβ synthesis when APP is overexpressed. However, it is unclear how this conclusion can be extrapolated to the disease or the process of normal aging, especially if we consider the lowering of cholesterol levels in a normal and pathological (in AD) aging brain [8]. A more likely scenario is the one in which a decreased cholesterol level in neuronal membranes, coupled with an increased Aβ production, a reduced Aβ degradation, and an enhanced inflammatory response, contributes to the progression of AD [22, 39]. It should be noted that treatment with statins has no significant beneficial effect in AD, even though statins significantly reduce plasma cholesterol levels [43]. Moreover, several studies have found cognitive deficits in response to statin treatments [44]; so, in January 2014 the U.S. Food and Drug Administration (FDA) issued a recommendation on the risks of statins. A promising finding is the use of phytosterols in AD, which supress Aβ production *in vitro *by inhibiting both the activity and expression of β- and γ-secretases, and BACE1 internalization into endosomes. The effect of phytosterols on Aβ processing may be associated with their stimulatory action on LX-receptors or the ability to accumulate in lipid rafts; the later facilitates the re-localization of APP and presenilin to a non-raft phase [45].

**ApoE and AD**

Low levels of ApoE-particles in the brain correlate with an increase in the risk of AD, but it is unclear whether this is associated with cholesterol transport. By interacting with receptors, ApoE triggers anti-apoptotic signaling pathways. ApoE binds to Aβ, then the complex passes through the BBB with the help of LRP, thereby lowering the concentration of Aβ in the brain. Aβ interaction with ApoE-particles is potentiated by sulfated derivatives of galactocerebrosides, the concentration of which in the brain is lower in AD [2, 46, 47]. An agonist of the nuclear retinoid X receptor rapidly increases the production of ApoE and promotes Aβ degradation, decreasing the formation of Aβ-plaques [48]. Oxysterol (24-OH Ch), which promotes the expression of ApoE, ABCA1, and ABCG1 via the stimulation of LX-receptors, has similar effects [1, 30].

There are three known human ApoE isoforms, which differ from each other only by one amino acid residue. The most common allele is *Apoε3, *while *Apoε4 *is detected only in 15–20% of the population and is considered a risk factor for AD with late onset. The probability of AD in individuals with one copy of *Apoε4 *is 4 times, and with two copies, 12–20 times higher than in carriers of the *Apoε3 *allele. The presence of *Apoε2*, conversely, hinders the progression of AD. Why *Apoε4 *provokes the disease has not yet been established [2, 8]. There are several possibilities [2, 5, 48-51]: 1) ApoE4 binding to Aβ is weaker, causing less efficient clearance; 2) ApoE4 is produced in smaller quantities, quickly decomposes, and does not form dimers that promote Aβ degradation; in contrast, Aβ in a complex with ApoE4 becomes resistant to degradation by neprilysin; 3) ApoE4 is less effective in maintaining axonal growth and the survival of neurons; 4) ApoE4 promotes the endocytosis of APP and BACE1 and their targeting to early endosomes, thereby increasing Aβ synthesis; 5) ApoE4 reduces the expression of reelin receptors in synapses, blocking its protective properties (*Fig. 2B*).

In AD, the C-terminal fragment of ApoE that promotes the accumulation of neurofibrillary bundles may appear in the brain. Cellular stress *in vitro *can trigger the fragmentation of ApoE with production of the toxic fragment. ApoE4 is more susceptible to cleavage, and expression of truncated ApoE4 leads to AD-like neurodegeneration [8, 52].

Variations in the other genes involved in the cholesterol metabolism are also risk factors for AD: for example, polymorphism of lipoprotein receptors (LRP1, LRP10, SorLA, ApoER2) and transporters (ABCA1, ABCA7, clusterin) genes [2]. LRP1 is involved in Aβ uptake and elimination, and a decrease in its expression contributes to Aβ accumulation. However, LRP1 increases the rate of endocytosis and directs APP into the lysosomes, which may upregulate Aβ synthesis [53]. A lower rate of endocytosis is typical for LRP1B; therefore, LRP1B inhibits the formation of Aβ [2]. SorLA/LR11, the level of which decreases in the late forms of AD, interacts with APP monomers, preventing their dimerization. This reduces APP cleavage by the γ- and β-secretases that prefer to use APP dimers as a substrate [54]. LRP10 and SorLA enhance APP traffic to the Golgi complex, where the secretases are less active [55]. Weak ABCA1 activity may contribute to AD, whereas its overexpression reduces the accumulation of Aβ. ABCA1 deficiency leads to the production of lipid-poor ApoE particles and a decrease in the amount of ApoE (by ~ 80%); additionally, cholesterol esters accumulate in the peripheral tissues [56].

**Synaptic pathology in AD**

Synaptic dysfunctions in AD represent the earliest events leading to cognitive deficit. At the early stages of AD, a decrease in glutamatergic transmission occurs in the cortex and hippocampus. The presynaptic events are the first to change, and the impairment of postsynaptic processes is recorded later. Long before the amyloid plaque formation, elimination of synapses and neuronal death, the synthesis of the key proteins of exo-endocytotic machinery (SNAP-25, synaptophysin, AP-2, AP-180, dynamin 1, synaptotagmin) decreases in the prefrontal cortex, and the first cognitive defects are observed [8, 57-59]. Despite the variety of the effects of ApoE4 gene variants – changes in the APP processing, decrease in Aβ clearance, synaptic plasticity interruptions – there is a common pathway for ApoE4 action associated with changes in endocytic recycling, probably through the reduction in the expression and activity of endocytotic proteins [51]. In patients with AD, early endosomes are 32 times larger in volume and the enlargement of endosomes begins before the manifestation of clinical symptoms in Apo*ε*4 carriers [60]. Levetiracetam, acting on the SV2A protein of synaptic vesicles, reverses the ApoE4-induced changes both in endosomal traffic and Aβ processing [51].

**Fig. 3:**
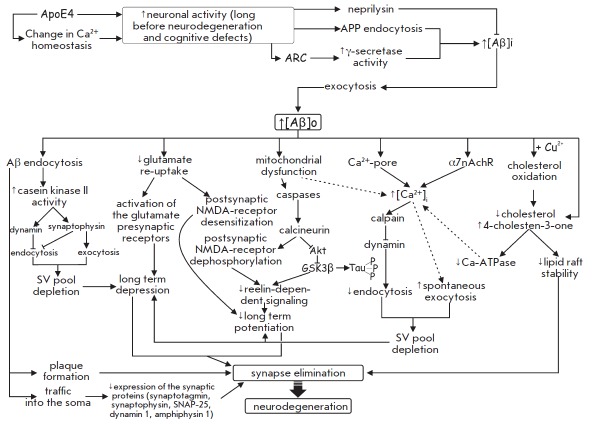
Pathways of amyloid peptide β (Aβ) action on synaptic transmission and plasticity. Role of neuronal activity in Aβ accumulation. See text for details.

High neuronal activity increases the production of Aβ in normal and pathological conditions (e.g., epilepsy). This is partly due to intense synaptic vesicle endocytosis, whereby APP molecules are captured in endosomes and are cleaved therein [61]. Also, soon after a burst of synaptic activity the early gene *Arc *is expressed and the Arc protein then increases the association of γ-secretase with APP in endosomes [62]. During vesicular exocytosis, the generated Aβ is released into the synaptic cleft, where it can regulate both neurotransmitter release and reception (*Fig. 3*). Synaptic activity can reduce the intraneuronal accumulation of Aβ by increasing the activity of neprilysin [63]. It is assumed that APP and Aβ are elements of “physiological” feedback, which controls synaptic transmission. Blocking Aβ production in young mice reduces their performance in memory tests [64]. Overproduction of Aβ may be caused by excessive/impaired synaptic activity. Individuals who carry mutations in presenilin 1 show an increased activation of the hippocampus 15 years before the onset of AD [65]. Enhanced expression/activity of ryanodine receptors in nerve terminals, causing an increased cytosolic Ca^2+^ and exocytosis, may occur before manifestations of histological and cognitive defects [59]. ApoE4 changes the brain activity in the early period: carriers of the *Apoε4 *allele have a higher activation of the hippocampus at rest and when performing memory tests [66]. ApoE4 interferes with reelin-dependent signaling, which is involved in the migration, maturation, survival of neuronal cells, and synaptic plasticity [2, 8]. ApoE4 suppresses the effects of reelin, because it decreases the number of available ApoE-receptors by preventing the return of the receptors to the plasma membrane after the endocytosis induced by binding to reelin and ApoE [50]. In addition, reelin signaling becomes susceptible to the toxic action of Aβ. Aβ via a mechanism involving mitochondrial dysfunction may activate caspase 3, which (1) stimulates calcineurin (phosphatase PP2B) and (2) cleaves the protein kinase Akt. Subsequently, PP2B dephosphorylates NMDA-receptors in the sites for Fyn phosphorylation and loss of Akt leads to disinhibition of GSK3β-kinase [67]. As a result, the long-term potentiation in response to the activation of ApoE receptors by reelin, which normally causes activation of Fyn and inhibition of GSK3β-kinase, is suppressed in the hippocampus [50]. Hyperactivity of GSK3β-kinase may be a factor that contributes to excessive phosphorylation of the tau protein, leading to the formation of neurofibrillary bundles, which disconnect from microtubules and may diffuse throughout the neuron [8, 67].

**The effects of the extra- and intracellular Aβ on synaptic transmission**

In synapses with weak activity, Aβ ([pM]) can activate presynaptic α7-nicotinic cholinergic receptors, promoting an increase in cytosolic Ca^2+^ and the release of a neurotransmitter. In high doses, Aβ ([nM]) can enhance the internalization of postsynaptic NMDA- and AMPA-receptors and long-term depression [58, 68]. By blocking the reuptake of glutamate, the elevated levels of Aβ lead to a decrease in the quantum size and a persistent increase in glutamate concentration in the synaptic cleft. Thus, postsynaptic NMDA-receptors become desensitized from the excessive stimulation while presynaptic NMDA and metabotropic glutamate receptors are still activated, causing long-term depression [57]. Aβ binds to presynaptically located P/Q-type calcium channels leading to the inhibition of the neurotransmitter release [69]. Aβ can form a Ca^2+^-pore, the entry of Ca^2+^ through which activates protease calpain that cuts endocytotic protein dynamin 1 [70]. The level of Cu^2+^ increases in AD, Aβ in a complex with Cu^2+^ acquires the ability to convert cholesterol into 4-cholesten-3-one, and its concentration is increased in AD [36]. The accumulation of 4-cholest-3- one can inhibit synaptic Ca^2+^-ATP-ase, disrupt the stability of lipid rafts, and depress neurotransmission [71]. Aβ may be involved in establishing the balance between silent and active synapses: “low activity” synapses increase their “job” in response to Aβ, while highly active synapses reduce it (*Fig. 3*). It is worth noting that nerve terminals in old animals are more susceptible to the negative effect of Aβ. These nerve terminals are characterized by a smaller total synaptic vesicle pool, weak mitochondria activity, and antioxidant capacity. Under these conditions, the inhibition of synaptic vesicle recycling induced by Aβ is substantially lower in the presence of exogenous antioxidants [72].

The severity of AD correlates with the presence of Aβ42 in neurons (especially in the neocortex), and the suppression of neurotransmission coincides with the accumulation of Aβ inside the nerve terminal, long before the appearance of extracellular plaques [28]. Aβ is trapped inside the endocytosed vesicles, and the presence of Aβ42 in these vesicles activates casein kinase 2, which by phosphorylation of dynamin and synaptophysin promotes the inhibition of endocytosis and exhaustion of synaptic vesicle pools after the synaptic activity [73]. Aβ absorbed into vesicles directly interacts with synaptophysin and disrupts the formation of a complex between synaptophysin and VAMP2, thereby increasing the number of primed vesicles and enhancing exocytosis [74]. However, after intensive exocytosis, endocytosis is weak, since the interaction of synaptophysin/VAMP2 is required for efficient endocytosis. Chronic administration of Aβ in non-toxic concentrations inhibits the glutamate release in the hippocampal nerve terminals by reducing the size of readily releasable and recycling pools [75]. Perhaps that also can be attributed to the fact that Aβ42 in endosomes are moved by axonal transport from nerve terminals to cell bodies, where Aβ42 suppresses the expression of exocytotic and endocytotic proteins: SNAP-25, synaptotagmin, synaptophysin, dynamin 1, and amphiphysin 1 [57, 58]. After endocytosis, Aβ can be directed towards multivesicular bodies, where Aβ forms fibrils that perforate the membranes, causing neuronal death. These fibrils subsequently form plaques [76]. In general, many studies indicate that endocytosis is the key event which is associated with the formation, elimination, and toxicity of Aβ.

## PARKINSON’S DISEASE (PD)

Parkinson’s disease (PD) is the second-most common neurodegenerative disease, which is characterized by tremor, slowed movements, rigidity, and cognitive impairment. As with AD, significantly fewer cases of PD are associated with mutations in specific genes, such as α-synuclein, parkin, LRRK2, PINK1, DJ-1, and ATP13A2. A peculiar feature of PD is the accumulation of α-synuclein in neurons as a part of protein inclusions, the so-called Lewy bodies. It affects dopaminergic neurons in the substantia nigra of the midbrain. It is worth noting that contemporary studies have revealed that 60% of patients with AD have deposits of α-synuclein in the amygdala, and that some patients with PD have an accumulation of Aβ in the brain [8, 19, 30, 77]. This suggests that specific pathways leading to the development of PD or AD converge, causing the appearance of common signs.

**Fig. 4:**
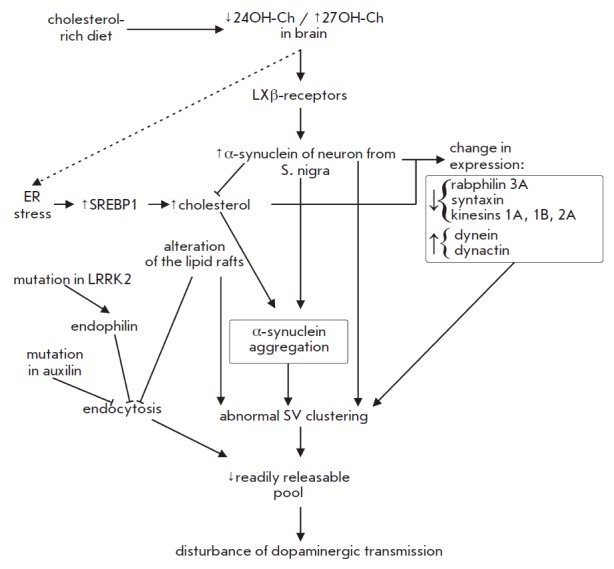
Relation between cholesterol, α-synuclein, and alterations in presynaptic events with dopaminergic neuron dysfunction in Parkinson’s disease. See text for details.

The role of cholesterol in PD remains controversial (*Fig. 4*). A study of lipid rafts isolated from the frontal cortex of subjects with early-stage PD showed a decrease in polyunsaturated fatty acids without changes in the content of cholesterol and sphingomyelin [78]. However, α-synuclein comprises two cholesterol-binding domains and membrane cholesterol affects its aggregation [41]. Theoretically, synuclein can disrupt the lipid raft integrity, by interacting with cholesterol [1]. Cholesterol depletion (using methyl-β-cyclodextrin) reduces the α-synuclein level in the membrane and its accumulation in neuronal bodies and synapses. Statins inhibit the aggregation of α-synuclein in a neuronal culture and the addition of exogenous cholesterol increases α-synuclein aggregation, which suppresses the growth of neurons [79]. Food deprivation (on the model of 3D5-cells) causes the aggregation of α-synuclein and apoptosis, which is associated with ER stress and SREBP1 activation, followed by an increase in cholesterol synthesis [80]. In PD, the concentration of some oxysterols increases in the brain in response to the overproduction of reactive oxygen species [81]. A cholesterol-rich diet reduces the ratio of 24OH-Ch : 27OH-Ch in the brain and increases the level of α-synuclein in substantia nigra without altering the cerebral cholesterol level. 27OH-Ch promotes and 24OH-Ch prevents an increase in α-synuclein concentration in a SH-SY5Y human neuroblastoma cell line. Moreover, 27OH-Ch exerts this effect through the activation of the LXβ- receptors that bind to the promoter of the α-synuclein gene [19].

Long before the neuronal death, the dopamine release is suppressed at the early stage of PD. Mutations and duplications/triplication of the α-synuclein gene causes PD with an early onset. α-Synuclein is concentrated in nerve terminals and binds to synaptic vesicles. Normally, synuclein is important for the clustering of vesicles in the proximity of the active zone, since it simultaneously interacts with the synaptobrevin 2 of one vesicle and the phospholipids of another vesicle, acting as a bridge. Mutation in the α-synuclein gene may reduce its vesicle-clustering ability, and its overexpression causes massive aggregation of synaptic vesicles, and eventually both lead to a decrease in the size of the readily releasable pool [82]. Overexpression of α-synuclein contributes to its intensive interaction with the membranes of synaptic vesicles and multivesicular bodies and disruption of their functioning [83]. Overexpression of mutant α-synuclein significantly alters the levels of the proteins involved in vesicular traffic and exocytosis (reduced quantities of rabfilin 3A, syntaxin, kinesins 1A, 1B, 2A, increased levels of dynein, dynactin 1) in the substantia nigra and the striatum [82]. A significant reduction in transcript levels (dynamin 2, AP-2, syntaxin-2, VAMP A, VAMP 4), implicated in vesicular cycling, was detected in the peripheral blood of patients at the first stage of PD [84].

In the case of hereditary PD, associated with a mutation in the *LRRK2 *gene (kinase containing leucine rich repeat), the traffic of synaptic vesicles is disrupted at the beginning of the disease. LRRK2 normally phosphorylates endophilin, thereby inhibiting its association with the membrane. At the same time excessive or insufficient activity of LRRK2 hinders synaptic vesicle endocytosis [85]. The juvenile form of PD is caused by mutations in the auxilin which participates in the uncoating of synaptic clathrin-coated vesicles.

## AUTISM SPECTRUM DISORDERS (ASD)

Autism spectrum disorders (ASD) are characterized by significant abnormalities in social interaction, difficulty in communication, and stereotypical behavioral patterns. These disorders may arise from genetic alterations, prenatal exposure to viruses and toxins, and interactions of the mother’s and fetus’ immune systems [30]. ASD are often associated with hereditary diseases, such as the Rett and fragile X syndromes, neurofibromatosis type 1, tuberous sclerosis, phenylketonuria, and the Smith-Opitz-Lemley syndrome.

Recently, new details have emerged concerning a relationship between cholesterol metabolism and the pathogenesis of some ASD. The Rett syndrome, which affects mainly women (1/10,000), is often associated with mutations in the X-linked gene of methyl-CpG-binding protein 2 (MeCP2). MeCP2 interacts with methylated DNA in the nucleus and recruits various transcription factors that regulate gene transcription, including those involved in cholesterol homeostasis. In the Rett syndrome, the levels of total cholesterol, the high- and low-density lipoproteins are increased, and the expression of scavenger receptor B1, responsible for the uptake of cholesterol, is reduced [86]. Expression of the genes involved in cholesterol metabolism was found to be slightly elevated in the brains of one-month-old mice with a mutation in Mecp2/Y. At the age of 2 months, these mice had an increased level of total cholesterol in the brain, but the production of cholesterol was suppressed, probably due to the activation of a negative feedback. As a result, by day 70 of postnatal development, the cholesterol concentration returned to its normal range. Reducing the production of cholesterol (mediated by a mutation in the gene *Sqle*/squalene epoxidase or by statins) in the mutant Mecp2/Y mice prevented a progression of the disease [87]. Treatment of women with the Rett syndrome with statins also improves their mental state. Therefore, early abnormalities of cholesterol metabolism in the Rett syndrome may contribute to the behavioral phenotype and a decrease in the survival rate [86]. It should be noted that statins also inhibit the synthesis of isoprenoid intermediaries (farnesyl pyrophosphate and ubiquinones), affecting such protein modifications as prenylation, which is important for the functioning of signaling proteins, such as Ras [88].

Statins are effective in the fragile X syndrome and type 1 neurofibromatosis [88]. The fragile X syndrome is one of the known causes (1/4,000) of mental retardation and autism. This syndrome occurs due to the expansion of CGG triplet repeats (greater than 200 repeats, full mutation) in the promoter of the *FMR1 *gene (fragile X mental retardation 1), which leads to hypermethylation and suppression of *FMR1 *gene transcription [30]. Reduced production of the FMR protein (RNA-binding protein that inhibits the translation induced by the activation of several receptors in dendrites) enhances the synthesis of some proteins involved in neurotransmission. Studies of animal models of the fragile X syndrome revealed an abnormally high activity of group I metabotropic glutamate receptors (mGR-I). Caveolin 1 and membrane cholesterol availability can regulate the traffic and signaling of these receptors [42]. The signaling mediated by mGR-I is attenuated in the neurons of mutant mice treated with statins. Additionally, such treatment reduces the abnormally increased protein synthesis and long-term depression in the hippocampus, audiogenic seizures, and visual cortex hyperexcitability.

ASD are closely related to synaptic dysfunction. Signs of ASD usually manifest themselves in early childhood, when sensory experiences modify and tune the excitation/inhibition balance; therefore, it is assumed that disruption of the glutamate/GABAergic transmission ratio may contribute to ASD. The synaptic theory of ASD began with the identification of a mutation leading to the disease in the gene for neuroligin, a postsynaptic cell-adhesion molecule. Then, it was found that many genes associated with predisposition to autism encode synaptic proteins [90]. Mutations in presynaptic cell-adhesion molecules, neurexins, inhibit synaptic vesicle exocytosis in the hippocampus and cause social behavioral anomalies. Dysfunction of the CASP2 protein, which regulates the release of electron-dense granules with neuropeptides (neurotrophin-3, brain-derived neurotrophic factor) and monoamines, increases the risk of autism [91]. Mutations in the *Mecp2 *gene reduce the levels of synaptic proteins, including synapsins, and the vesicular glutamate transporter. There is also a decrease in the expression of the GABA-synthesizing enzyme GAD and the size of the neurotransmitter quantum in GABA synapses [92]. The fragile X syndrome involves downregulation of the GABA-A receptor α5- and γ-subunits and tonic currents through receptors composed of these subunits. Mice without reelin (normally expressed in cortical interneurons) exhibit a ASD phenotype and decreased GABA spillover [93]. Genetic polymorphism of exocytotic protein SNAP-25, mutations in presynaptic synapsin 1, 2, and the active zone protein RIMS3, which affect the neurotransmitter release, are associated with the probability of ASD. Mutations in the postsynaptic proteins IL1RAPL1 and SynGAP1 involved in synapse formation are also related to ASD [91].

## Abbreviations

Aβ: amyloid peptide β
ABC: ATP-binding cassette transporters
ApoE: apolipoprotein E
AD: Alzheimer’s disease
PD: Parkinson’s disease
OH-Ch: hydroxycholesterol
BBB: blood-brain barrier
LRP: LDL-receptor related protein
LX-receptor: Liver X receptor
ASD: autism spectrum disorders
CYP46A1: cholesterol 24-hydroxylase
CYP7B1: oxysterol 7α-hydrolase
ER: endoplasmic reticulum
